# Change in quality of life and self-esteem in a randomized controlled CBT study for anxious and sad children: can targeting anxious and depressive symptoms improve functional domains in schoolchildren?

**DOI:** 10.1186/s40359-021-00511-y

**Published:** 2021-01-21

**Authors:** Kristin D. Martinsen, Lene-Mari P. Rasmussen, Tore Wentzel-Larsen, Solveig Holen, Anne Mari Sund, Marit Løtveit Pedersen, Mona Elisabeth S. Løvaas, Joshua Patras, Frode Adolfsen, Simon-Peter Neumer

**Affiliations:** 1 Regional Centre for Child and Adolescent Mental Health, Eastern and Southern Norway, RBUP, region East and South, Oslo, Norway; 2 Department of Psychology, University of Oslo, Oslo, Norway; 3 The Regional Centre for Child and Youth Mental Health and Child Welfare – North, Faculty of Health Sciences, UiT The Arctic University of Norway, Tromsø, Norway; 4 Regional Centre for Child and Youth Mental Health and Child Welfare, Medical Faculty, NTNU, Norwegian University of Science and Technology, Trondheim, Norway; 5 Department of Child and Adolescent Psychiatry. St. Olav’s University Hospital, Trondheim, Norway; 6 Institute of Psychology, NTNU, Norwegian University of Science and Technology, Trondheim, Norway

**Keywords:** Children, Adolescent, Quality of life, Self-esteem, Anxiety, Depression, CBT

## Abstract

**Background:**

Quality of life and self-esteem are functional domains that may suffer when having mental problems. In this study, we examined the change in quality of life and self-esteem when targeting anxious and depressive symptoms in school children (8–12 years) using a CBT-based transdiagnostic intervention called EMOTION, Kids Coping with anxiety and depression. The aim of this study was to investigate quality of life and self-esteem in children with elevated levels of anxious and depressive symptoms, and further if the EMOTION intervention could influence these important functional domains.

**Methods:**

The study had a clustered randomized design (cRCT), where *N* = 795 children recruited from 36 schools participated. The children were included based on self-reports of anxious and depressive symptoms. Schools were the unit of randomization and were assigned to intervention or control condition. Children in the intervention condition received the 10-week EMOTION intervention. Mixed effects models were used to take account of the possible clustering of data. Separate models were estimated for the dependent variables.

**Results:**

Children with elevated levels of anxious and depressive symptoms reported lower levels of quality of life and self-esteem compared to normative samples, with girls and older children reporting the lowest levels. For both genders and older children, a large and significant increase in quality of life and self-esteem was found among the children who received the intervention compared to the children in the control condition. Children in the intervention group reporting both anxious and depressive symptoms showed a significantly larger increase in both quality of life and self-esteem compared to the controls. Reductions in quality of life and self-esteem were partially mediated by reductions in symptoms of anxiety and depression.

**Conclusions:**

Participating in an intervention targeting emotional symptoms may have a positive effect on quality of life and self-esteem in addition to reducing anxious and depressive symptoms. Improved quality of life may increase the child’s satisfaction and subjective perception of wellbeing. As low self-esteem may lead to anxious and depressive symptoms, improving this functional domain in children may make them more robust dealing with future emotional challenges.

*Trial registration* NCT02340637, retrospectively registered

## Background

It is well documented that anxiety and depression are highly prevalent and often comorbid in youth [1] and can lead to significant functional impairments in many domains such as academic achievement, peer and family relations [2–4]. Even more prevalent are children who have anxious and depressive symptoms that do not qualify for a diagnosis, yet still have functional reductions that are comparable to children with clinical disorders [5]. Two important domains that may suffer when having anxious and depressive symptoms are self-perceived quality of life and self-esteem.

Quality of life may be defined as the subjective perception of well-being and satisfaction which is best evaluated by the child according to his or her own experience within several life domains [6]. Several studies have reported the relation between quality of life and child psychiatric disorders [6–9], concluding that children with mental health problems report lower quality of life compared to healthy children. In addition, children with a physical disorder or internalizing disorders have lower quality of life compared to children with externalizing disorders [8]. Hence, mental health problems interfere with important aspects of the child’s well-being such as peer-relations, school performance and family functioning, which again affects quality of life [10]. In a community sample of youth aged 12–17 years [11], those with moderate anxiety scores had corresponding quality of life scores similar to Scandinavian youth with OCD diagnosis [9]. A young person’s perception of quality of life can provide important information regarding how mental health problems affects him or her. It may therefore be important to examine quality of life when evaluating interventions aiming to reduce symptoms of anxiety and depression, especially regarding how change in specific symptoms relate to quality of life. In fact, relatively low reductions in symptom levels may translate to large gains in self-reported quality of life, indicating that the relationship may not be a one-for-one proposition. For this reason, some have argued that change in self-perceived quality of life may be an important additional estimate of change than symptom reductions [12]. Self-reported quality of life could also be an indication of the severity regarding internalizing problems in addition to symptom levels [13], and at least it indicates a subjective experience of someone with anxious and depressive symptoms.

Self-esteem is often defined as an individual’s global evaluation of his or her worth as a person [14]. High self-esteem is connected to better social relationships [15] and academic achievement [16]. High self-esteem also seems to serve a protective role for the development of mental health problems, and early research found that high self-esteem could buffer for anxiety [17]. The association between low self-esteem and anxiety and depression is later confirmed [e.g., 18–20]. In a recent study of Norwegian treatment seeking adolescents, high self-esteem at baseline predicted a reduction in symptoms of both anxiety and depression [21], and the researchers suggested that high self-esteem could act as a buffer under stress through better use of coping strategies. Low self-esteem on the other hand is related to several negative outcomes, including poor psychological health [22].

Several theoretical models concerning the link between self-esteem and depression have been suggested [23]. The vulnerability model assumes that the level of self-esteem causally influences the onset and maintenance of depression [24], but not vice versa [25, 26]. The meta-analysis by Sowislo and Orth [27] and Orth and Robins [23] supported the association between low self-esteem and negative affectivity, suggesting that low self-esteem contributes to depression. Self-esteem is, however, unstable during adolescence. This instability in self-esteem also implies that self-esteem is changeable [28], and interventions that aim to change low self-esteem may be effective [14]. The normative course is that self-esteem decreases across adolescents’ years [29], and that there are some gender differences, where boys seems to report higher on self-esteem than girls [e.g., 14]. Hence, a decline in self-esteem may increase the risk of depression, while an increase may reduce the risk [14]. Furthermore, Steiger, Allemand [14] found that change in self-esteem had significant effects on depressive symptoms two decades later, where youth who had decreasing self-esteem during adolescence, reported more depressive symptoms later in life. Change in self-reported levels of self-esteem may therefore have important implications for the young person, and interventions with the potential to improve self-esteem are warranted.

In the present study the objective was to examine if self-reported quality of life and self-esteem would change when targeting anxious and depressive symptoms evaluating the transdiagnostic prevention program EMOTION *“Coping Kids” Managing Anxiety and Depression* [30]. EMOTION is an indicated preventive program based on cognitive behavioral therapy (CBT) aiming to reduce anxious and depressive symptoms in school children aged 8 to 12 years with elevated symptoms levels. Recruiting symptomatic children in this age range is important, because early intervention can prevent the symptoms developing into clinical disorders. School-based universal prevention programs that target anxious and depressive symptoms in youth generally produce small positive effects [31]. Similar results are found across population based, indicated and selective prevention programs [32–35]. To our knowledge, there is little information regarding how a preventive intervention targeting anxious and depressive symptoms in school children can potentially affect functional domains such as quality of life and self-esteem.

In the current study, we analyzed information regarding the children’s self-perceived quality of life and self-esteem when targeting their anxious and depressive symptoms. We first hypothesized that children with elevated levels of anxious and depressive symptoms, would report lower quality of life and self-esteem than the normative group, and that children would report different levels of quality of life and self-esteem depending on age and gender. In addition, we hypothesized that change in symptoms of anxiety and depression would mediate the relationship between the intervention and quality of life and self-esteem. Lastly, we expected a statistically significant change in quality of life and self-esteem in the at-risk groups with children reporting mixed symptomatology (both anxious and depressive symptoms) compared to children reporting anxious or depressive symptoms only.

## Method

The present study was part of a large multisite study with a clustered randomized design (cRCT) following the Extended Consort requirements [37]. The study was an effectiveness study where *N* = 1686 children from 36 schools in Norway were recruited and underwent screening from spring 2014 until summer 2016. Children with elevated levels of anxious and/or depressive symptoms (*N* = 795) participated in groups conducted by mental health professionals as part of their ordinary work. Change in anxious and/or depressive symptoms were the primary outcomes of the original study. The main effects of the intervention om these primary outcomes were positive and were published in a brief report [36]. Schools were the unit of randomization. Allocation of the schools to (a) intervention condition (EC) or (b) control condition (CC) involved pairing schools based on geographical area, school-size and demography, and then randomly assigning schools to one condition at the beginning of the study. Randomization was conducted by coin flip with one senior researcher and a project staff member. Schools in both conditions could provide any psychosocial programs (e.g. anti-bullying programs) while participating in the study. In all participating schools (both EC and CC), teachers were provided with a 3-h lecture about anxious and sad children. The lecture included information on how to support the children, and how the children could seek assistance for their difficulties, e.g. talking to the school health nurse.

For a detailed description of the multisite study, see the publication of the protocol [38].

The Regional Committees for Medical and Health Research Ethics (2 013/1909/REK Sør-Øst) approved the study.

### Participants

Children in this study (*N* = 795, 58.0% girls) were between 8 and 12 years of age (*M* = 9.64, SD = 0.93) and 97.7% were of Norwegian, Nordic or West-European origin. The children included in analyses were in the following grades: 3^rd^ grade *n* = 35 (4.4%), 4^th^ grade *n* = 291 (36.6%), 5^th^ grade *n* = 363 (45.7%) and 6^th^ grade, *n* = 106 (13.3%). There were no significant gender and age differences between the youth in the EC and CC condition preintervention.

Analysis of missing values showed that the same individuals had not completed the quality of life and self-esteem measures at T2 (*N* = 102, 12.8%). There was no evidence of a relationship between missing at T2 and gender (p = 0.498), while there was evidence of an increasing relationship between age and missing at T2 (p < 0.001). In grade 3, the percentage of missing was 2.9%, increasing to 25.5% missing in sixth grade.

**Fig. 1:**
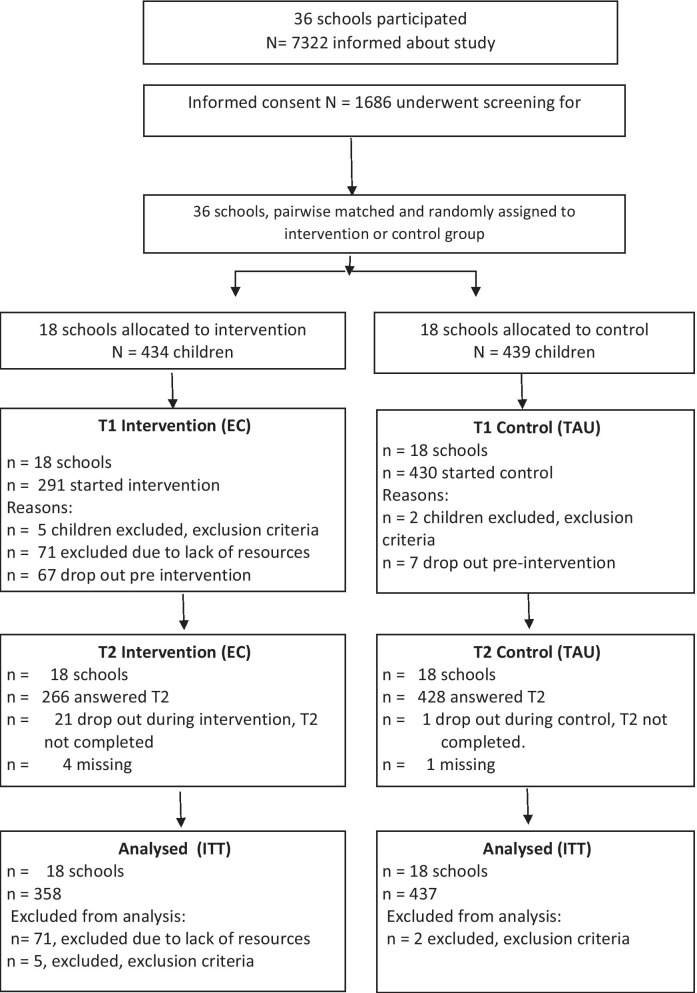
Flowchart showing flow of children in the main study in accordance with Consort guidelines

See also Consort statement showing the flow of participants in the study (Fig. 1).

### Procedure

Children were recruited from both urban and rural areas at seven sites and a stepwise recruitment procedure was used. First all the children and their parents in the 3^rd^ to 6^th^ grades at the participating schools were informed about the study. When parents provided informed consent, children were screened for anxious and depressive symptoms. Children scoring 1 SD or more above population means on self-reported symptoms of anxiety, depression or both were invited to participate in the study [39]. Exclusion criteria were mental retardation, pervasive developmental disorders, and otherwise not being able to benefit from a group intervention based on recommendations by teachers at the school or the child’s parents. While few children were excluded based on teachers and parents’ recommendation (*N* = 7), reasons provided for such exclusion were severe cognitive or developmental challenges or that a child being bullied would be placed in the same group as the bully.

### The EMOTION intervention

The transdiagnostic EMOTION intervention is a CBT-based program, which targets anxious and depressive symptoms in schoolchildren and their parents within the same manuals. Children met in child group sessions twice a week for 10 weeks, while the parents met in groups for seven sessions with children attending four of these. Child groups had a maximum of seven participating children, while both parents were encouraged to meet in parent groups.

The manuals [40] were developed to guide group leaders, while parents and children were provided with workbooks. The first half of the program is dedicated to skill building and the second half focus on behavioral experiments, cognitive restructuring and enhancing self-esteem. Improving low self-esteem was specifically targeted in the ten last sessions with a focus on developing a differentiated self-perception. Parents and teachers contributed by providing input to positive aspects of the child as son/daughter and as a student.

Important components of the intervention aimed at reducing anxious and depressive symptoms and improving self-esteem are presented in Table 1. As can be seen, parents learned many of the same skills as the children to support the children in their process.

**Table 1:** The EMOTION intervention

Child and parent sessions with objectives		
Child sessions/strategies	Objectives/mechanisms targeted	Parent sessions/strategies
Motivation/goal setting, group cohesion, closing (S: 1, 3, 19, 20)	Collaborative relationship/focused approach	Motivation/goal setting/closing (S: 1, 7)
Coping through pleasant events/Emotion focused coping (S: 2, 4)	Emotion understanding and emotion regulation	Identification of feelings/Emotion focused coping (S: 4)
Psychoeducation: Identification of feelings, how thoughts influence feelings (S: 3, 6)		
Problem-solving/Cognitive restructuring (S: 5, 7, 8, 9 & 10–18)	Information processing errors	Problem-solving/Cognitive restructuring (S: 5, 6)
Exposure & Behavioral activation (S: 10–18)	Withdrawal/behavioral learning	Exposure & Behavioral activation/support (S: 4, 5)
Building a differentiated self-schema (S: 12–16)	Self-esteem	Positive parenting/ positive reinforcement/support (S: 2, 3)
	Parental behavior	Positive parenting/ positive reinforcement/coping modeling/support (S: 2, 3)

S = Session The EMOTION intervention has 20 child sessions, and 7 parent group meetings

Trained group leaders delivered the intervention during school hours. They were recruited from primary health services and mental health care, including primarily school health nurses and psychologist from the pedagogical/psychological services They were trained in the program during a two-day training course involving experimental learning and roleplay. The group leaders also received weekly supervision.

The effect of the transdiagnostic prevention program EMOTION has been evaluated in a cRCT both short and long-term [36, 41]. Children’s self-report of anxious and depressive symptoms indicated a small, but significant, effect at post-intervention [36]. At 12-months follow-up, children reported a small but significant reduction of anxious symptoms, while parents reported a decline in depressive symptoms [41].

### Measures

The secondary outcome measures of the effectiveness study [36], the Kinder Lebensqualität Fragebogen (KINDL) [42](www.kindl.org.) and the Beck Youth Inventory; BYI-II [43] was analyzed in this study to assess quality of life and self-esteem.

The KINDL was developed for epidemiological use in children and adolescents aged 4–16 years and has been used in several clinical and epidemiological studies [44]. It consists of 24 items and measures physical and emotional wellbeing, self-esteem, and social functioning (family, friends, and school) on a five-point scale from 1 “*never*” to 5 “*all the time*”. The KINDL questionnaire is analyzed by adding the item responses marked on each sub-scale, transforming the scores to standardized scores enabling comparisons to be made with norm data [45]. Higher scores indicate better quality of life. Internationally, a mean score of 81.9 (*SD* = 9.07) is reported from a normative sample of schoolchildren (4^th^ grade) (*N* = 846) [45]. For adolescents, a sum score above 70 has been suggested as indicative of being in good health [46]. Similar sum scores were also reported in youth aged 7 – 17 years (mean age was 13), in a Norwegian control sample (*M* = 69.72, SD = 12.4) [9].

In a study with children aged 8–16 years, a Norwegian version of the KINDL showed satisfactory internal consistency and test–retest reliability of the KINDL total quality of life scale [47]. Cronbach’s alpha in the current study was 0.87.

Self-esteem was assessed using the Beck Self-Concept Inventory for Youth (BSCI-Y II), which is a subscale of the Beck Youth Inventory; BYI-II [43]. The BSCI-Y II measures self-concept in children between 7 and 18 years using 20 items, and is considered useful for screening in schools [43]. The self-concept inventory measures the child’s perception of self, body image, competence, and relation to others. Statements are rated on a four-point scale, “1” for “*never*”, 2” for “*sometimes*”, “3” for “*often*” and “4″ for “*always*”. The total sum score based on all items was used in the analysis [43]. Norms are based on gender and three age groups. The inventory also has Norwegian norms (*N* = 600) [48]. In the age range 7–10 years of age, the following means and standard deviations are reported for Norwegian children: boys: *M* = 41.62 (SD = 7.63), girls: *M* = 40.94 (SD = 7.39). Furthermore, the internal reliability of the Norwegian version was high (Cronbach’s alpha in the 0.8–0.9 range). Cronbach’s alpha in the current study was 0.90.

Children were recruited into the study based on the children’s self-reported symptoms of anxiety using the Multidimensional Anxiety Scale for Children (MASC-C) [49] and symptoms of depression using the Short Mood and Feelings Questionnaire (SMFQ) [50]. Both measures have good psychometric properties [49–51].

### Statistical analysis

Because of the possible clustering of data, children attending the same school could be assumed to be more similar than children attending different schools and the assumption of independence of observations may be violated [52]. Therefore, we used mixed effects models, including random effects at the school level, the individual level, and within the individual to account for this issue. Mixed effects models give valid inference for missing at random in dependent variables. The relationships of missing values at T2 with quality of life, self-esteem, gender, and age, were investigated using Chi-square tests.

Separate models were estimated for the dependent variables KINDL and BSCI-Y II. Random structures were investigated for stability and simplified when necessary [53]. Fixed effects included a time by randomization group (condition) interaction, and analyses were adjusted for gender and age group (i.e., children in 3^rd^ and 4^th^ grade in the age range 8–10 were classified as younger; children in 5^th^ and 6^th^ grade in the age range 10–12 were classified as older). Subgroup analyses were performed within each gender and age group. Furthermore, we performed analysis on the groups reporting anxiety only, depression only and combined anxiety and depression group, referred to as at-risk groups. Intention to treat analysis was used [54].

We also examined whether the changes in self-esteem and quality of life were mediated by changes in symptoms of anxiety or depression by using causal mediation analysis [55] based on linear regression analyses with change in self-esteem and quality of life as dependent variables. With causal mediation analysis indirect (mediated) effects and direct effects are estimated in a setting where causality of the relationships has been assumed. The indirect effect (the average causal mediation effect, ACME) is the effect mediated via changes in symptoms of anxiety or depression, while the average direct effect (ADE) refers to other effects of the intervention (differences in changes in self-esteem and quality of life between the intervention and control condition).

First the regression results were checked for similarity with results of the corresponding mixed effects models. We then assessed the potential direct and indirect effect of changes in symptoms of anxiety and depression on changes in self-esteem and quality of life. Gender and age were entered as adjustment variables to reduce the risk for unmeasured confounding in the causal mediation analysis. Causal mediation analysis is based on an untestable assumption of no unmeasured confounding and includes a procedure to check for the sensitivity of the results for this assumption. A parameter rho, the correlations between error terms of regressions for the outcome and the mediator, measures deviations from the assumption. If the sign of the effect of the intervention is unchanged for a large rho interval the sensitivity is considered as small.

The statistical program IBM SPSS (version 22) was used for descriptive analyses. Estimation of mixed effects models were conducted using the R (The R Foundation for Statistical Computing, Vienna, Austria) package nlme. Causal mediation analyses was performed used the R package mediation [56].

## Results

Mean scores on primary outcome measures of quality of life and self-esteem as reported by children pre- and post-intervention are shown in Table 2.

**Table 2:** Means and standard deviations of quality of life (KINDL) and self-esteem (BSCY-II) pre- and post-intervention

Children													
		Pre-intervention						Post-intervention					
		Intervention (*N* = 358)			Control (*N* = 437)			Intervention (*N* = 265)			Control (*N* = 428)		
Measure		*N*	Mean	SD	*N*	Mean	SD	*N*	Mean	SD	*N*	Mean	SD
KINDL	All	358	60.79*	13.49	437	63.08	12.13	265	66.43	14.13	428	65.93	13.99
	Boys	137	61.88*	14.50	197	66.11	11.84	96	66.50	15.71	192	68.04	13.60
	Girls	221	60.11	12.81	240	60.59	11.81	169	66.38	13.20	236	63.64	13.86
	3rd and 4th gr	142	64.96*	13.41	184	66.45	12.57	115	66.82	14.54	182	66.95	13.49
	5th and 6th gr	216	58.04	12.85	253	60.63	11.19	150	66.13	13.85	248	64.63	14.15
BSCY-II	All	358	34.82*	9.77	437	36.68	9.28	265	38.86	10.40	428	38.35	10.26
	Boys	137	35.52*	10.15	197	38.76	9.32	96	39.37	11.14	192	39.60	10.65
	Girls	221	34.39	9.52	240	34.39	8.91	169	38.75	9.98	236	36.75	9.60
	3rd and 4th gr	142	36.87*	10.57	184	38.93	9.07	115	39.43	11.22	182	39.81	9.66
	5th and 6th gr	216	33.48	8.98	253	35.05	9.10	150	38.42	9.74	246	36.71	10.36

*N* = 795. gr. = grade. KINDL = Kinder Lebensqualität Fragebogen, BSCY-II = (Beck youth inventory-II-self-concept scale)

*Significant difference between intervention- and control condition at T1

There were significant pre-intervention differences in mean scores between the intervention and the control condition for all children on the measure of quality of life (KINDL) and for self-esteem (BSCY-II), where the control-group reported higher levels of quality of life and self-esteem than the intervention-group did.

### Change in quality of life and self-esteem

We first ran the analyses with schools included. This multilevel model was, however, unstable for some subgroups for gender. In these cases, confidence intervals for random effects were not computable, gave extremely low or high confidence limits, or were inconsistent across different reference categories for time or randomization group (Pinheiro & Bates, 2000). The models were re-run without the school level and are reported below.

### Quality of life (KINDL)

**Fig. 2:**
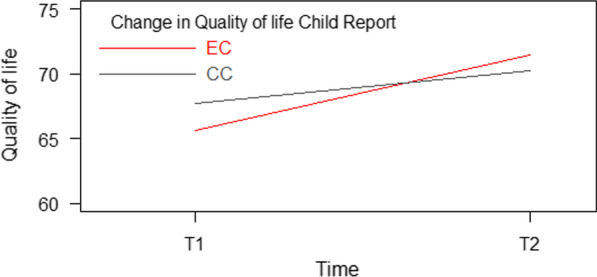
Significant interaction – quality of life. Note: Kinder Lebensqualität Fragebogen (KINDL) (Ravens-Sieberer et al., 2013). EC = Emotion condition, CC = Control condition

The interaction of Time and Condition was significant (*p* = 0.001), indicating a larger increase in self-reported quality of life in the intervention condition (EC) compared to the control condition (CC). In the EC, there was an increase in quality of life of 5.83 (8.9%) points. In CC, the increase was 2.57 (3.8%) points. Post intervention, the EC reported higher levels of quality of life than the CC, but the difference was not significant, see also Fig. 1. We found a significant difference in the two conditions for gender where girls reported 3.48 lower scores than boys, and where older children reported 4.21 lower scores on quality of life than younger children. The results are presented in Table 3 and graphically in Fig. 2.

**Table 3:** Model based estimates for development in child self-reported changes of quality of life (KINDL) and self-esteem (BSCY-II)

Quality of life (KINDL)	Child report		
	Coefficient	95% CI	*p *Value
Condition by time interaction	− 5.19		
EC vs CC at T1	− **1.95**	− 3.79, 0.11	0.037
EC vs CC at T2	1.30	− 0.66. 3.27	0.194
T2 vs T1, EC	**5.83**	4.33, 7.33	< 0.001
T2 vs T1, CC	**2.57**	1.36, 3.79	< 0.001
Girls vs boys	− **3.48**	− 5.12, − 1.84	< 0.001
Older vs younger	− **4.21**	− 5.85, − 2.58	< 0.001
Self-esteem (BSCY-II)			
Condition by time interaction			
EC vs CC at T1	− **1.63**	0.27, 2.99xx	0.019
EC vs CC at T2	1.07	− 2.53, 0.38xx	0.149
T2 vs T1, EC	**4.05**	2.94, 5.16	< 0.001
T2 vs T1, CC	**1.35**	0.45, 2.26	0.003
Girls vs boys	− **2.37**	− 3.58, —1.15	< 0.001
Older vs younger	− **3.09**	− 4.30, —1.88	< 0.001

N = 791. KINDL = Kinder Lebensqualität Fragebogen, BSCY-II = (Beck youth inventory-II-self-concept scale). Significant results are shown in bold

EC = Emotion Condition, CC = Control condition, T1 = pre-intervention, T2 = post-intervention

In subgroup analyses by gender adjusted for age, there was a significant interaction of Time by Condition for girls *F*(403,1) = 5.66, *p* = 0.018. Girls in the EC had an increase of 6.09 (4.20–7.97), *p* < 0.001 points on the KINDL, while girls in CC had an increase of 3.06 (− 5.07—0.11), *p* < 0.001. For boys, there was also a significant interaction of Time by Condition *F*(286,1) = 4.62, *p* = 0.033. Boys in the EC had an increase of 5.36 points (2.86–7.85), *p* < 0.001 points on quality of life, while boys in CC had an increase of 1.99 (0.16–3.81), *p* = 0.033.

Subgroup analyses of older and younger children adjusted for gender, indicated a significant interaction of Time by Condition for older children *F*(394,1) = 11.02, *p* = 0.001, but not for younger children *F*(295,1) = 1.62, *p* = 0.204. In the EC, older children had an increase in quality of life score of 8.41 (6.42–10.40), *p* < 0.001 and younger children had an increase of 2.36 points (0.14–4.57), *p* = 0.037. In CC, the increase in quality of life scores for older children was 4.09 (2.48–5.70), *p* < 0.001, and for younger children the increase was only 0.51 (− 1.28–2.31), *p* = 0.574.

### Self-esteem (BSCI-Y)

**Fig. 3:**
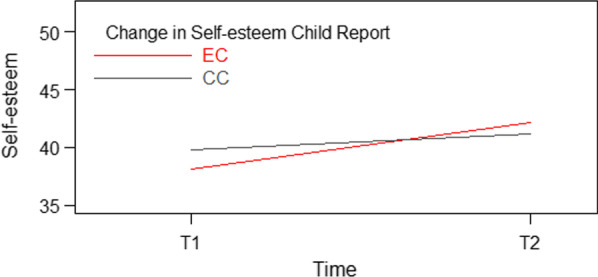
Significant interaction – self-esteem. Beck youth inventory-II-self-concept scale (BSCY-II) [43]. EC = Emotion condition, CC = Control condition

For self-esteem, (Table 3), the interaction of Time and Condition was significant, *p* < 0.001 indicating a larger increase in self-esteem in the EC compared to the CC. In the intervention group, self-esteem increased with 4.05 (10.6%) points. In CC, the increase was 1.35 (3.4%) points. At post intervention, the difference between the groups was not significant, (see Fig. 3). For gender and age there were also significant differences; girls scored 2.37 points lower than boys, and older children scored 3.09 points lower than younger children.

In gender subgroup analyses, we found a significant Time by Condition interaction for girls, *F*(1,403) = 6.4, *p* = 0.012 and for boys, *F*(1,286) = 6.64, *p* = 0.011).

Subgroup analyses by age revealed a significant interaction for older children, *F*(1,394) = 12.19, *p* < 0.001. Older children in the EC had an increase of 4.9 points on the measure of self-esteem whereas younger children in CC had an increase of only 1.68 points. For younger children, the interaction was not significant *F*(1,295) = 2.92, *p* = 0.089.

### AT-RISK groups—change in quality of life and self-esteem

For the at-risk group where children reported both anxious and depressive symptoms the Time and Condition interaction was significant, *p* = 0.004. For the at-risk groups reporting only anxious or only depressive symptoms, the interaction of Time and Condition for quality of life was not significant (Table 4).

**Table 4:** At-risk groups: model based estimates for development in child self-reported changes of quality of life (KINDL) and self-esteem (BSCY-II)

Quality of life (KINDL)		Anxious symptoms only (N = 164)			Depressive symptoms only (N = 234)			Anxious and depressive symptoms (N = 397)		
		Coefficient	95% CI	*p *Value	Coefficient	95% CI	*p *Value	Coefficient	95% CI	*p *Value
Condition by time interaction	EC vs CC at T2	− 0.40	− 4.09, 3.28	0.829	− 3.04	− 6.55, 0.47	0.089	− 4.19	− 7.03, − 1,36	**0.004**
Self-esteem (BSCY-II)										
Condition by time interaction	EC vs CC at T2	− 4.25	− 7.75, − 0.76	**0.018**	− 2.74	− 5.37, − 0.11	**0.041**	− 2.02	− 3.97, − 0.06	**0.044**

KINDL = Kinder Lebensqualität Fragebogen, BSCY-II = Beck youth inventory-II-self-concept scale

Regarding self-esteem there was a significant Time and Condition interaction for all groups: anxious symptoms only, *p* = 0.018, depressive symptoms only, *p* = 0.041, both. Anxious and depressive symptoms combined, *p* = 0.044.

### Causal mediation analysis

For self-esteem the indirect effects mediated via changes in symptoms of anxiety were large (46.24%) (average causal mediated effect, ACME = 1.28 (0.783, 1.843), p < 0.001 and via changes in depression (25.2%) (ACME = 0.69 (0.107, 1.294), *p* = 0.026). The direct effects of the intervention were also substantial, for anxiety (average direct effect, ADE = 1.50 (0.107, 1.294), *p* = 0.044) and for depression (ADE = 2.05 (0.665, 3.36), *p* = 0.004). Sensitivity analysis indicated that the changes in self-esteem for the indirect effects were not so robust towards deviations from the assumption of no unmeasured confounding (reversed sign for ρ between -0.3 to -0.2 for changes in anxiety and between -0.4 to -0.3 for changes in depression). For changes in anxiety the direct effect on self-esteem was also not so robust (reversed sign for ρ between 0.3 and 0.4), while the direct effect of changes in depression was robust, (ρ between 0.7 and to 0.8).

For quality of life the analysis also indicated large indirect effects (46.5%) mediated via changes in symptoms of anxiety (ACME = 1.28 (0.787, 1.871), *p* < 0.001) and depression (24.0%) (ACME = 0.67 (0.102, 1.30), *p* = 0.004). Furthermore, the analysis also indicated large direct effects for anxiety (ADE = 1.43 (− 0.08, 2.87), *p* = 0.062, and for depression (ADE = 2.09 (0.785, 3.382), *p* = 0.004).

Again, sensitivity analysis showed that the indirect effect on quality of life mediated via changes in symptoms of anxiety and depression were not so robust (reversed sign for ρ between -0.3 to—0.2 for anxiety, and 0.4 to 0.3 for depression) and similarly for the direct effect of anxiety (reversed sign for ρ between 0.3 and 0.4). The direct effect on quality of life for depression was robust (ρ between 0.7 and 0.8) indicated that the results for the direct effects were robust towards deviations from the assumption of no unmeasured confounding.

## Discussion

In accordance with our hypothesis in the current study, children reported lower quality of life and self-esteem than what is found in normative samples [48, 57]. Results also indicate significant differences between gender and age groups, which also supports our hypothesis. In addition, we identified a larger and significant increase in quality of life and self-esteem among those who received the intervention compared to the control condition at post intervention. These findings also supported our hypothesis. Both genders as well as older children reported a significant increase compared to children in the control condition. Children in all at-risk groups experienced a significant positive change in self-reported self-esteem. For quality of life, however, only the group reporting both anxious and depressive symptoms, showed a significant positive change after the intervention, providing a partial support of our hypothesis. Examining possible mechanisms of change, we found large indirect effects via the mediators (changes in symptoms of anxiety and depression), and also large direct effects of the intervention. The mediated effects amounted to between 24.0 and 46.5%, with changes in symptoms of anxiety showing the strongest indirect effect. Hence our hypothesis that the increase in self-reported quality of life and self-esteem mainly was mediated by reductions in symptoms of anxiety and depression was partially supported.

Investigating the level of quality of life and self-esteem reported in this group of symptomatic school children, yielded some interesting results. Quality of life was reported almost two standard deviations below the mean in normative samples [57] and lowest for girls and older children. Actually, children in this community sample of at risk-children scored as low on the quality of life measure as a Norwegian sample of pediatric OCD patients [9]. This supports previous findings, that children with mental challenges are reporting much lower quality of life compared to children with other difficulties [6]). Quality of life has been argued to play an important role in defining health problems [58], and even in this sample of non-clinical children, the children’s well-being is seriously affected. Screening for children’s self-perceived quality of life could therefore allow for early detection of health care needs. The quality of life reported is also an indication of the burden associated with specific problems, where the strongest associations have been observed between mental health problems and quality of life [59].

Self-esteem was reported approximately one standard deviation lower by the children in this sample compared to normative groups [48], and again girls and older children reported the lowest levels. This also supports previous findings about the link between low self-esteem and negative affectivity [27], and that youths with mental health problems have lower self-esteem as opposed to children without such problems [60].

While quality of life and self-esteem are expected to decrease during adolescence [28, 45], results from the current study indicate that even younger children with anxious and depressive symptoms have lower levels in these important functional domains compared to normative groups at the same age. Identifying anxious and sad children at an early stage and encouraging them to learn coping strategies may be an important way to improve domains important to children’s well-being.

Findings from the current study also showed that older children (5^th^ and 6^th^ grade) scored significantly lower on quality of life and self-esteem than younger children (3^rd^ and 4^th^ grade). This could be related to the child`s natural development, and that self-esteem in particular is viewed as a malleable construct which decreases when children approaches adolescence [14]. However, the older children in this study are still relatively young (around 11–12 years of age), and most have not reached their most vulnerable stage in adolescence yet [61]. Gender differences with girls reporting lower quality of life and self-esteem, is also reported in earlier research where increasing gender role pressures has been suggested as a possible cause [14, 58].

The “side-effects” of increasing quality of life and self-esteem when addressing and improving anxious and depressive symptoms in a group-based study, is interesting. Firstly, these findings provide an external validation of the main findings related to the effectiveness study of the EMOTION program where the same children reported significant reductions in anxious and depressive symptoms [36].

These results are somewhat different from the findings reported in the meta-analysis by Taylor and Montgomery [62] who reported that the effect of CBT on global self-esteem among youth did not reach significance at post-treatment. They are however in concordance with the proposition that self-esteem is changeable and that interventions targeting self-esteem may be effective [14, 28].

Results from the mediation analysis gives an indication of possible mechanisms at work, suggesting that the self-reported changes in self-esteem and quality of life both can be partially attributed to mediation via the changes in the levels of anxiety and depression. Reduced symptom levels may have enhanced children’s experience of themselves. Gaining new positive experiences when meeting previously avoided situations and perhaps thinking more realistically when challenged may have contributed to the considerable indirect effects. Furthermore, the children may have improved both their coping strategies and confidence as a result of participating in the intervention. Mechanisms such as group participation and getting support from other children may also have contributed to the substantial direct effect.

It thus seems possible to improve the children’s perception of themselves and their quality of life by learning how to cope with challenging emotions and situations. The active focus on establishing a more differentiated self-schema and the suggested relationship between cognition and self-esteem [62] could also influence the negative view of oneself by accessing more realistic thoughts. Such tasks are emphasized in the latter part of the intervention.

Children in the intervention condition reported a higher increase in quality of life than the control condition did, and within the intervention condition, the older children reported the largest change. Actually, children who participated in EMOTION groups reported an 8.9% increase in quality of life corresponding to an increase of 5.8 point, and older children reported the largest change with of 8.41 points. While still not in the normative range as reported on German children [45], the scores are approaching the quality of life scores displayed in a Norwegian general population sample of children in the same age range [9, 63]. Hence, this improvement could bring them within the population range on quality of life. The importance of personal, social and family-based resources for quality of life in youth has been emphasized [58], and this is exactly what is provided through the EMOTION intervention. In a group with peers, the children learn skills to cope better with challenging situations and parents participate in separate groups to translate the skills into the home environment. To study if these skills hold, or even may improve in the long run, remains still to be seen.

The increase in self-esteem in the intervention condition compared with the control condition, especially for older children, have implications as it is important to prevent a further development of depressive symptoms into depressive disorder. The fact that low-self-esteem is proposed as a risk-factor for developing depression at a later stage [23] and that high self-esteem may have a buffering effect for the development of anxiety [17] are important reasons for targeting the child’s global evaluation of him- or herself in an intervention. Based on this rationale, screening for low self-esteem could also help identify children at risk for developing internalizing difficulties.

Henriksen and colleagues [21] suggested that high self-esteem could act as a buffer under stress by the youth using active problem-solving strategies instead of avoidant strategies. The EMOTION intervention has several sessions devoted to learning problem-solving strategies and emphasizes the use of behavioral experiments, where you approach situations that are normally avoided (e.g. calling/visiting a friend or being present in class). Focusing on building a positive self-schema is an important part of the EMOTION program and should be highlighted in continued use of the program. This focus promotes establishing a positive and differentiated evaluation of oneself. Having a more positive evaluation of oneself is suggested to counteract the symptoms of mental health problems [21]. Running the program in primary schools may be especially important, as schools are an important arena for young children’s development, including their self-esteem [64]. Furthermore, improved self-esteem where the children are approaching scores within the normative range [48] may be important to achieve before the transition to secondary school where new challenges may increase stress in youth.

Older children did seem to benefit more from the intervention in relation to quality of life and self-esteem compared to younger children. This may imply that the EMOTION program targets some important issues in this age group, and that reaching the children before problems become too rigid and less changeable is important. That older children benefitted more could also indicate that maturation and increased cognitive levels is an advantage, perhaps enabling them to use more active coping strategies such as problem solving and cognitive restructuring improving their self-schemas. This is also suggested in the literature [62, 65].

The importance of targeting self-esteem was also supported by looking closer at the at-risk groups. Results indicate that there was a significant positive change in self-reported self-esteem in all at-risk groups (anxious symptoms only, depressive symptoms only and anxious and depressive symptoms). Thus, spending time on strengthening the children`s self-esteem is an important asset of this transdiagnostic program, especially considering the potential impact it could have later in life [14, 25].

For quality of life, there was only a significant association for the children who reported both anxious and depressive symptoms in the at-risk groups. Other studies have indicated that quality of life is more influenced when there are comorbid conditions, and that duration of symptoms may be associated with lower quality of life [9]. Having both anxious and depressive symptoms may be an indication of the child having developed symptoms over a longer period of time, as anxious symptoms at any given time predict depressive symptoms appearing at a later stage [66]. As previously suggested [13], the severity and level of internalizing problems may be an indicator of how much impact the mental health problems have on the child`s life quality.

Summing up, it seems possible to improve the child’s quality of life and perception of themselves by learning how to cope with and handle challenging emotions and situations, which is an important ingredient in the EMOTION group intervention.

### Strengths and limitations

The cluster randomized, controlled design, large sample size, together with low attrition and rate of missing data, were strengths of the current effectiveness study. This study was part of a larger intervention study conducted in real-world settings with group leaders already working in different school or mental health services. A high number of participants from different parts of the country, both urban and rural areas, is another possible strength. Furthermore, the possible clustering of the data was accounted for in the analyses. Another strength of the study is the use of well-established instruments to measure both quality of life and self-esteem as well as internalizing symptoms. A rigorous design combined with a high-quality implementation of the program (e.g. training, supervision, and ongoing booster sessions) ensures that the program was delivered similarly in all the municipalities, thus strengthening the results.

However, some limitations are worth mentioning. Given that this was an indicated study, and the recruitment was based on self-selection, the sample is not representative for the full at-risk child group in the population. Self-recruitment might lead to some children excluding themselves due to a reluctance to participate in groups (e.g. social anxiety) or other reasons (e.g. missing information given about the study). There was also a significant pre-intervention difference between the intervention and the control condition, which may indicate that the groups were systematically different. This could be due to the restricted cluster randomization, however, further investigations indicated that the differences were random.

## Conclusion

Participating in the EMOTION program targeting anxious and sad children gave a larger increase in quality of life and self-esteem in the intervention condition compared to the control condition. Furthermore, we found strong indirect effect via changes in symptoms of anxiety and depression and also large direct effects of the intervention. Quality of life and self-esteem seems to be in a reciprocal association with symptoms of anxiety and depression, and low levels of quality of life and self-esteem might lead to mental disorders. Targeting sadness and anxiousness early in life is imperative, as it might not just reduce symptoms of internalizing problems, but also improve their quality of life and self-esteem.

More specifically, low self-esteem seems to follow the trajectory of anxious and depressive symptoms. As such, focusing on self-esteem may be an important part of an intervention for young schoolchildren with anxious and/or depressive symptoms. Building a positive self-schema could help enhance the children`s global evaluation of themselves, thus, making them more capable of dealing with emotional challenges.

How anxiety and depression relate to quality of life and self-esteem may therefore provide valuable information regarding the impact of mental health problems on children’s well-being. Furthermore, impairment in quality of life and self-esteem associated with specific mental health problems can, together with the burden of the problems to society [67], be used by policy makers to set priorities. The findings are also important for future studies on children’s global functioning regarding mental health development.

## Data Availability

The datasets generated during and/or analyzed during the current study are available from the corresponding author on reasonable request.

## Abbreviations

MASC-C: Multidimensional anxiety scale for children questionnaire
SMFQ: Short mood and feelings questionnaire
BYI-II: Beck youth inventory-II questionnaire, self-concept scale
KINDL: Kinder Lebensqualität Fragebogen Questionnaire
EC: EMOTION condition
CC: Control condition
Gr: Grade level
